# The Effect of Cannabidiol on Cancer-Pathway Genes in Doxorubicin-Sensitive and Resistant Breast Cancer Cells

**DOI:** 10.3390/ph19040615

**Published:** 2026-04-14

**Authors:** Kezban Uçar Çifçi, Ayşe Büşranur Çelik, Ebru Güçlü, Nisanur Şahinoğlu, Levent Gülüm, Emir Çapkınoğlu, Yusuf Tutar

**Affiliations:** 1 Division of Basic Sciences and Health, Hemp Research Institute, Yozgat Bozok University, Yozgat 66900, Türkiye; kezban.u.cifci@bozok.edu.tr (K.U.Ç.); ebru.guclu@bozok.edu.tr (E.G.); 2 Division of Molecular Biology and Genetics, Faculty of Hamidiye, Institute of Health Sciences, University of Health Sciences, Istanbul 34668, Türkiye; aysebusranur_celik25@erdogan.edu.tr; 3 Department of Basic Medical Sciences, Division of Biochemistry, Faculty of Medicine, Recep Tayyip Erdoğan University, Rize 53020, Türkiye; yusuf.tutar@erdogan.edu.tr; 4 Faculty of Molecular Biology, Yozgat Bozok University, Yozgat 66900, Türkiye; 16107123015@ogr.bozok.edu.tr; 5 Department of Crop and Animal Production, Mudurnu Süreyya Astarcı Vocational School, Bolu Abant İzzet Baysal University, Bolu 14030, Türkiye; leventgulum@ibu.edu.tr; 6 Department of General Surgery, School of Medicine, Acibadem Mehmet Ali Aydinlar University, Istanbul 34752, Türkiye; 7 Molecular Oncology Program, Health Sciences Institutes, Recep Tayyip Erdogan University, Rize 53100, Türkiye; 8 Molecular Medicine Program, Health Sciences Institutes, Recep Tayyip Erdogan University, Rize 53100, Türkiye; 9 Recep Tayyip Erdogan University Training and Research Hospital, Rize 53100, Türkiye

**Keywords:** cannabidiol, apoptosis, breast cancer, doxorubicin resistance

## Abstract

**Purpose**: Cannabidiol (CBD) is a primary bioactive, non-intoxicating cannabinoid found in the cannabis plant. Studies have shown that CBD causes anticancer activity by inhibiting the expression of growth factors and inducing apoptosis, leading to cell cycle arrest. In this study, we aimed to determine how CBD influences the expression of genes that affect cancer pathways in doxorubicin-sensitive (MCF-7) and doxorubicin-resistant (MCF-7/Adr) breast cancer cells. **Materials and Methods**: IC_50_ concentrations of CBD in MCF-7 and MCF-7/Adr cell lines were determined by the MTT cell cytotoxicity assay. RNA isolation and subsequent cDNA synthesis were performed for qPCR experiments with the determined IC_50_ values. The effects of CBD on the cell cycle and apoptosis were studied using flow cytometry. IC_50_ values of CBD were determined in MCF-7 and MCF-7/Adr breast cancer cell lines at eight different concentrations and at three different incubation periods (24 h, 48 h, and 72 h) with different doses. RT-qPCR was used to investigate the molecular mechanisms underlying the expression of genes involved in cancer pathway analysis. **Results**: Treatment with CBD at concentrations of 17.57 μM (MCF-7) and 11.41 μM (MCF-7/Adr) for 48 h decreased colony formation, induced apoptosis, and inhibited cell invasion in both cell lines. In addition, we observed significant alterations of angiogenesis, apoptosis, cell cycle, cellular senescence, DNA damage and repair, epithelial-to-mesenchymal transition, hypoxia, metabolism, telomeres, and telomerase in both cell lines. **Conclusions**: Our research indicates that CBD could be an effective natural bioactive compound for breast cancer treatment, inhibiting tumor cell proliferation and inducing apoptosis.

## 1. Introduction

Breast cancer (BC) is the most common malignancy in women worldwide. It is the second leading cause of cancer-related deaths. The disease is complicated by genetic and phenotypic heterogeneity, meaning that each patient requires a unique treatment. The gene expression profile and the presence or absence of surface receptors significantly impact the understanding of the biological heterogeneity of breast cancer, which categorizes them into four molecular subtypes (luminal A, luminal B, HER2, and triple-negative BC (TNBC)) [1]. Patients with breast cancer are classified as estrogen receptor α (ERα) positive or negative depending on the ERα status of the tumor, and 70% of patients are ERα positive, meaning that the growth and development of their tumors depend on estrogen [2].

There is a significant difference in chemotherapy sensitivity between Estrogen Receptor-Alpha-Negative and Estrogen Receptor-Alpha-Positive breast tumors. In patients with ER+/PR+ breast cancer, endocrine hormone therapies are commonly used as primary systemic therapies, complementing surgery. These include ovarian function suppression, selective estrogen receptor modulators, selective estrogen receptor downregulators, and aromatase inhibitors [3]. Chemotherapy, including doxorubicin/Adriamycin, paclitaxel, docetaxel, cyclophosphamide, and carboplatin, alone or in combination, is commonly used for neoadjuvant or adjuvant treatment of breast cancer, to downstage tumors or as a standard-of-care regimen for aggressive and early stage disease [4].

Despite significant advances in cancer treatment, doxorubicin, a drug with high therapeutic efficacy, is still in use for the treatment of breast cancer and other cancers.

Doxorubicin (DXR) is a chemotherapeutic drug, part of the anthracycline family, which is used to treat breast cancer. However, it has been shown that DXR can induce drug resistance and even tumor growth, resulting in poor patient prognosis and survival Although various mechanisms have been investigated, DXR resistance remains a significant and unresolved problem in the treatment of cancer patients. Studies have reported that the interaction between signaling pathways may promote DXR resistance by inducing proliferation, promoting cell cycle progression, and inhibiting apoptosis [5]. In 1986, Batist et al. established the MCF-7/Adr adriamycin-resistant cell line by incubating MCF-7 cells with increasing concentrations of the anthracycline antibiotic [6]. In cancer research, MCF-7/Adr cells are widely used as a model of multidrug-resistant MCF-7 cells [7]. Even though there has been a lot of research on doxorubicin resistance in breast cancer, we still do not know all the details about the specific genes and pathways involved in this process.

The plant Cannabis sativa has been used medicinally for several thousand years. More than 540 metabolites contribute to its therapeutic properties, and CBD is one of the key phytocannabinoids. Research has indicated that CBD has a broad range of therapeutic effects and is linked to cancer treatment [8]. Several studies have investigated the effects of CBD on breast cancer models, including MCF-7 cells. For example, Schoeman et al. showed that CBD decreases the viability of MCF-7 breast cancer cells and induces apoptosis in a dose-dependent manner [9]. In addition, in vivo studies support the antitumor potential of CBD in breast cancer models, highlighting its ability to modulate tumour growth and associated molecular pathways. These findings support the relevance of CBD as a potential therapeutic agent in breast cancer and provide a basis for further investigation [10]. These encompass the suppression of tumor cell proliferation and metastasis, as well as the initiation of autophagy or apoptosis. CBD can alter the tumor microenvironment, reducing cytokine secretion from cancer cells. CBD has influence on this process through cannabinoid receptors, mainly cannabinoid receptor 1 (CB1) and cannabinoid receptor 2 (CB2), as well as other signaling pathways including transient receptor potential vanilloid 1 (TRPV1), G protein-coupled receptor 55 (GPR55), and peroxisome proliferator-activated receptor gamma (PPARγ). CBD can alter the tumor microenvironment, reducing cytokine secretion from cancer cells [11]. Doxorubicin resistance is a significant barrier to effective breast cancer treatment; examining the therapeutic potential of CBD in both sensitive and resistant cell models is crucial. In this study, we aimed to determine the therapeutic efficacy of CBD that affects cancer pathways in doxorubicin-sensitive (MCF-7) and doxorubicin-resistant (MCF-7/Adr) breast cancer cells and to evaluate its effects on cancer-related pathways associated with drug resistance.

## 2. Results

### 2.1. Cannabidiol Inhibits the Proliferation of Breast Cancer Cells

**Figure 1:**
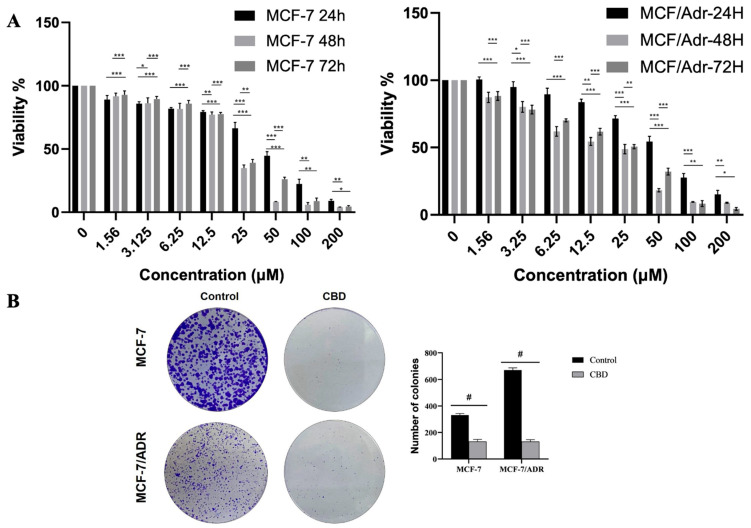
Cell viability (**A**) and inhibition of colony formation (**B**) by CBD in MCF-7 and MCF-7/Adr cells (Magnification: 10×). MCF-7, MCF-7/Adr cells were seeded into 96-well plates, and cells were treated with different concentrations (0–200 µM) of CBD for 24, 48, and 72 h. The survival rate of cells treated with CBD was measured by the MTT method. To determine the time-dependent effect of CBD, the % viability values determined for 24, 48, and 72 h for each dose were analyzed with one-way ANOVA followed by post hoc Tukey test (*p* < 0.05 *, *p* < 0.01 **, *p* < 0.001 ***, and *p* < 0.0001 #).

The effects of CBD on the viability of MCF-7 and MCF-7/Adr cells were evaluated using the MTT assay (Figure 1). As shown in Figure 1A,B, CBD treatment inhibited cell viability in both MCF-7 and MCF-7/Adr cell lines in a dose- and time-dependent manner. The IC_50_ values of CBD in MCF-7 cells after 24, 48, and 72 h of incubation were determined to be 30.37 ± 1.05 µM, 17.57 ± 1.56 µM, and 20.48 ± 1.25 µM, respectively. The IC_50_ values of CBD in MCF-7/Adr cells after 24, 48, and 72 h of incubation were determined to be 38.97 ± 0.12 µM, 11.41 ± 1.69 µM, and 16.68 ± 1.63 µM, respectively. These findings indicate that CBD exerted dose- and time-dependent inhibition in both cell lines. According to the colony formation capacity analysis, the number of colonies in cells treated with CBD at 17.57 μM (MCF-7) and 11.41 μM (MCF-7/Adr) decreased significantly compared with the control group (*p* < 0.0001 #).To determine the selectivity of cannabidiol (CBD) toward cancer cells, the same concentrations were applied to CCD-1072Sk normal fibroblast cells. The IC_50_ value in CCD-1072Sk cells was calculated as 45.36 ± 1.42. The selectivity index (SI), calculated as the ratio of the IC_50_ value in normal CCD-1072Sk cells to that in cancer cells, was determined to be 2.58, indicating a markedly higher sensitivity of cancer cells to CBD treatment.

### 2.2. CBD Induces Apoptotic Cell Death and Suppresses the Invasion of Breast Cancer Cells

**Figure 2:**
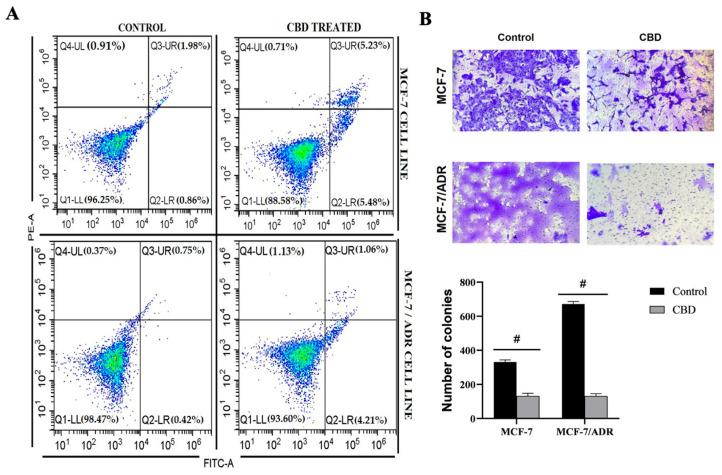
CBD induces apoptosis and suppresses invasive capacity in MCF-7 and MCF-7/Adr breast cancer cells. (**A**) CBD could effectively induce apoptosis in MCF-7 and MCF-7/Adr cells. After 48 h of treatment with CBD, the apoptosis rate of MCF-7 and MCF-7/Adr cells was detected by Annexin V-FITC/PI double staining. Data were analyzed with a One-Sample Binomial Test (*p* < 0.0001 #). (**B**) Images of invaded cells in control and CBD-treated groups; the number of invaded cells was counted (Magnification: 10×). Data were analyzed with one-way ANOVA followed by post hoc Tukey test (*p* < 0.0001 #).

FITC Annexin V analysis results showed an increase in the number of apoptotic cells in breast cancer cells treated with CBD. The rates of early and late apoptotic cells in MCF-7 cells treated with 17.57 μM CBD were 5.48% and 5.23%, respectively. On the other hand, in MCF-7/Adr cells treated with 11.41 μM CBD, the percentages of early and late apoptotic cells were 4.21% and 1.06%, respectively. CBD increases the number of apoptotic cells in MCF-7 compared with MCF-7/Adr. These results suggested that CBD triggered both early and late-stage apoptosis in breast cancer cells (*p* < 0.0001) (Figure 2A). Additionally, MCF-7/Adr cells treated with 11.41 μM CBD led to a significant increase in the number of necrotic cells compared to 17.57 μM CBD-treated MCF-7 cells (*p* < 0.0001) (Figure 2A).

Using a Matrigel invasion chamber, the effect of CBD on breast cancer cell invasion was assessed. CBD significantly decreased cell invasion of breast cancer cells after 48 h at doses of 17.57 μM (MCF-7) and 11.41 μM (MCF-7/Adr) compared to the control (*p* < 0.0001 #) (Figure 2B).

### 2.3. Cell Cycle

**Figure 3:**
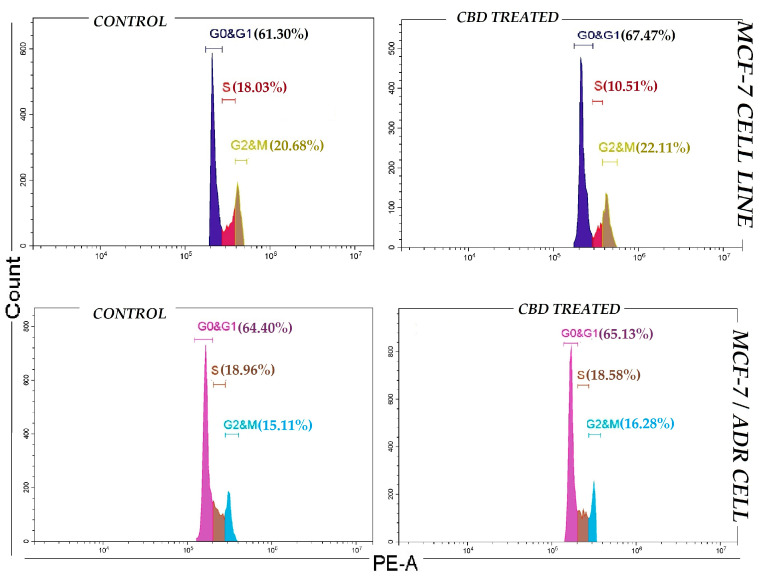
Evaluation of cell cycle in breast cancer cells (MCF-7 and MCF-7/Adr) treated with CBD after 48 h.

As shown in Figure 3, CBD increases the G0/G1 phase and induces cell cycle arrest in the S phase in MCF-7 cells depending on time. Our results showed that MCF-7 cells were arrested at G0/G1, compared with the control group. In contrast, CBD induces cell cycle arrest in the G0/G1 and G2/M phase of the MCF-7/Adr cell lines. This arrest was also confirmed by array analysis, which corroborated the cell cycle experiments.

### 2.4. RT-qPCR and Gene Enrichment

**Figure 4:**
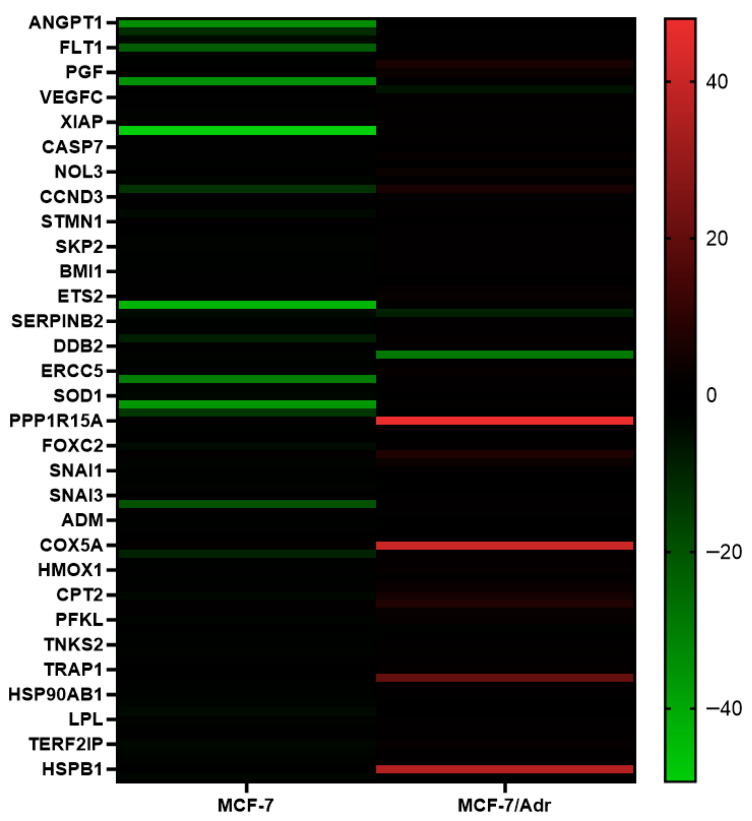
Heatmap of differential gene expression in MCF-7 and MCF-7/Adr cells after 48 h CBD treatment. The results were analyzed using the 2^−ΔΔCT^ method. Gene expression changes are visualized from red (high expression) to green (low expression). The heatmap was generated using GraphPad Prism version 8.0.2.

The effects of CBD treatment on cancer-related pathway genes in MCF-7 and MCF-7/Adr cells were investigated using RT-qPCR analysis. Total RNA was isolated 48 h after CBD treatment, and RT-qPCR was performed using the genes listed in Supplementary Table S1. The results were analyzed using the 2^−ΔΔCT^ method. CBD treatment led to differential gene expression changes in MCF-7 and MCF-7/Adr cells (Supplementary Tables S2 and S3, and Figure 4).

In MCF-7 cells, CBD treatment led to a decrease in the expression of angiogenesis-related genes ANGPT2, FLT1, and KDR; anti-apoptotic genes BIRC3 and XIAP; DNA damage repair-associated genes LIG4, POLB, SOD1, and TBX; cellular stress-related genes EPO and CA9; cell cycle-related genes CCND2, CCND3, CDC20, E2F4, MKI67, SKP2, AURKA, WEE1, and ETS2; energy metabolism-associated genes ACLY, ACSL4, LDHA, SLC2A1, CPT2, PFKL, UQCRFS1, GPD2, GUSB, and LPL; and telomere function and structure-related genes TNKS2, PINX1, TINF2, TERF1, TEP1, TERF2IP, and TNKS. In MCF-7/Adr cells, CBD treatment decreased the expression of angiogenesis-related genes FLT1 and TEK, while increasing the expression of apoptosis-related genes APAF1, CASP9, and BIRC3.

**Figure 5:**
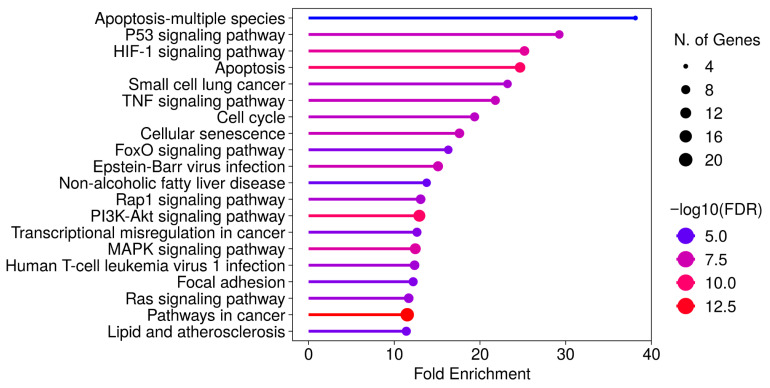
KEGG pathway enrichment analysis of differentially expressed genes in MCF-7 cells after CBD treatment. Pathway enrichment was conducted using the ShinyGO platform. Pathways were selected based on false discovery rate (FDR) values and ranked according to fold enrichment scores. (Accessed 30 June 2025.)

**Figure 6:**
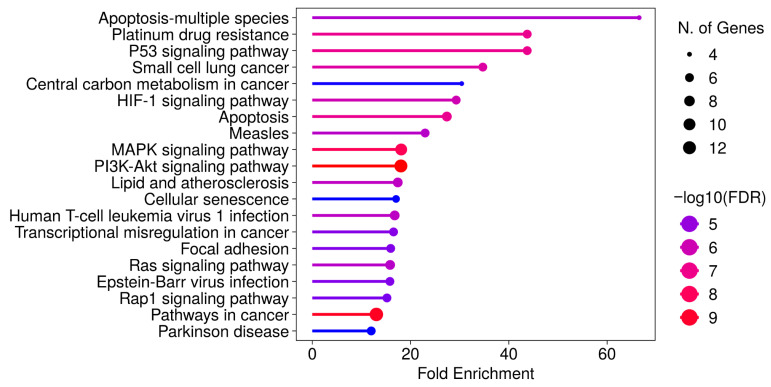
KEGG pathway enrichment analysis of differentially expressed genes in MCF-7/Adr cells after CBD treatment. Pathway enrichment was conducted using the ShinyGO platform. Pathways were selected based on false discovery rate (FDR) values and ranked according to fold enrichment scores. (Accessed 30 June 2025.)

Differentially expressed genes (≥2-fold upregulation or ≤0.5-fold downregulation) were identified, and KEGG pathway enrichment analysis was performed using the ShinyGO 0.82 platform. Despite differences in pathway ranking, similar enrichment patterns were observed in both cell lines (Figure 5 and Figure 6, Supplementary Tables S4 and S5). Commonly enriched pathways in both MCF-7 and MCF-7/Adr cells included p53, HIF-1, PI3K-AKT, MAPK, RAP1, and RAS signaling pathways, as well as pathways associated with apoptosis, senescence, and transcriptional misregulation in cancer. In addition to these, KEGG analysis of MCF-7 cells revealed enrichment in the TNF signaling pathway and cell cycle regulation, whereas in MCF-7/Adr cells, central carbon metabolism in cancer was also identified.

### 2.5. Gene-Metabolite Interactions

**Figure 7:**
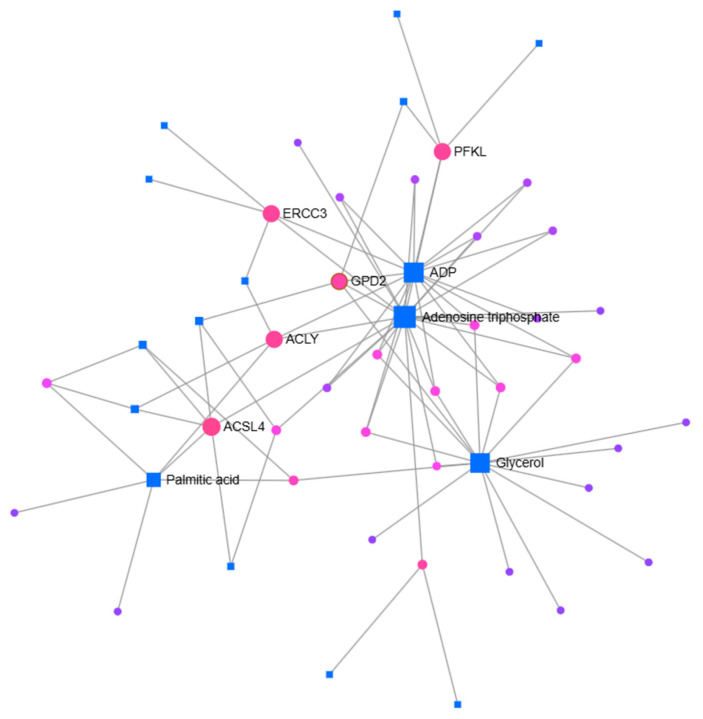
Network analysis of gene–metabolite associations in MCF-7 cells following cannabidiol treatment. Significantly differentially expressed genes were identified by RT-qPCR, and their associated metabolites were determined using the EnrichR platform with the Metabolomics Workbench Metabolites 2022 database. The interactions between these genes and metabolites were analyzed using MetaboAnalyst 6.0. Nodes represent genes (circles) and metabolites (squares), while edges indicate predicted associations. (Accessed 30 June 2025.)

**Figure 8:**
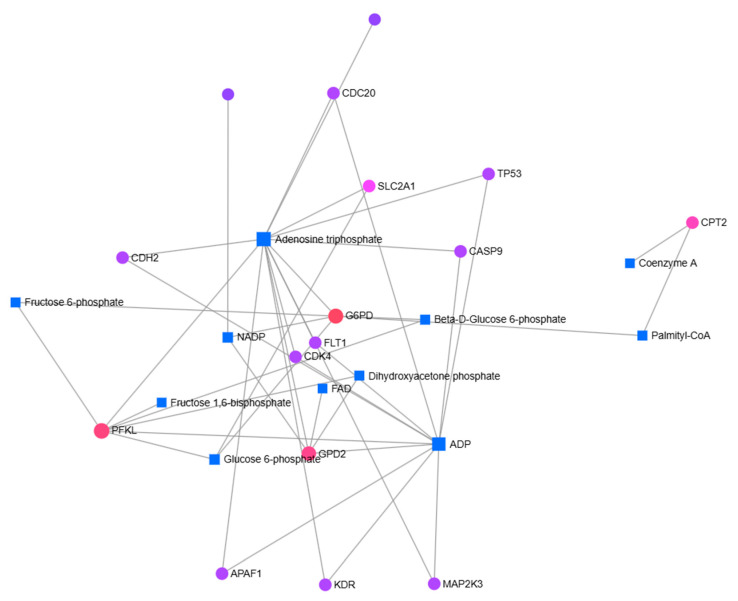
Network analysis of gene–metabolite associations in MCF-7/Adr cells following cannabidiol treatment. Significantly differentially expressed genes were identified by RT-qPCR, and their associated metabolites were determined using the EnrichR platform with the Metabolomics Workbench Metabolites 2022 database. The interactions between these genes and metabolites were analyzed using MetaboAnalyst 6.0. Nodes represent genes (circles) and metabolites (squares), while edges indicate predicted associations. (Accessed 30 June 2025.)

To determine gene–metabolite associations, metabolites related to significantly differentially expressed genes were identified using the EnrichR platform with the Metabolomics Workbench Metabolites 2022 database (Supplementary Tables S6 and S7). The relationships between the identified genes and metabolites were further analyzed using the MetaboAnalyst 6.0 database (Figure 7 and Figure 8). In MCF-7 cells, the genes ERCC3, PFKL, GPD2, ACLY, and ACSL4 were found to be associated with metabolites including adenosine diphosphate (ADP), adenosine triphosphate (ATP), palmitic acid, and glycerol (Figure 7). For MCF-7/Adr cells, the genes CDC20, CDH2, PFKL, SLC2A1, TP53, CASP9, FLT1, CDK4, APAF1, KDR, MAP2K3, and G6PD were associated with metabolites such as ADP, ATP, NADP, FAD, fructose 6-phosphate, glucose 6-phosphate, dihydroxyacetone phosphate, beta-D-glucose 6-phosphate, and palmitoyl-CoA (Figure 8).

## 3. Discussion

For over three decades, cancer therapy has relied extensively on natural compounds with broad pharmacological activity and proven clinical benefits. Cannabidiol (CBD), a non-psychoactive phytocannabinoid, has emerged as a promising anticancer molecule due to its ability to modulate cell survival, apoptosis, metabolism, and angiogenesis [12,13]. In this study, the aim was to evaluate the antitumor properties and transcriptional and cancer-related pathway-level effects of CBD in doxorubicin-sensitive (MCF-7) and doxorubicin-resistant (MCF-7/Adr) breast cancer cells, using various in vitro assays to assess its potential as a multitarget chemotherapeutic and chemosensitizing agent. Our study found a significant difference in the CBD-induced antitumor effect between the MCF-7 and MCF-7/Adr cell lines. These findings support prior studies showing that CBD reduces cell viability, induces apoptosis, and suppresses invasion in various breast cancer cells. The response varies by cell type, dose and exposure time but our results confirm the antiproliferative and pro-apoptotic profile of CBD. CBD treatment affects dose- and time-dependent cell viability in both cell lines, with IC_50_ values of 17.57 μM for MCF-7 and 11.41 μM for MCF-7/Adr after 48 h, comparable to those reported in other epithelial cancer models [14,15]. Treatment of both cell lines with the IC_50_ dose of CBD resulted in the suppression of cell proliferation, colony formation, and invasion as well as the induction of apoptosis, which increased by 10.71% in MCF-7 cells and by 5.27% in MCF-7/Adr cells, by simultaneously suppressing inhibitors of apoptosis (XIAP, BIRC3) and inducing pro-apoptotic mediators (BAX, BCL2L11/BIM, TP53). In one study, CBD inhibited cell proliferation and colony formation in SGC-7901 cells, upregulating p53 and Bax and downregulating p21 and Bcl-2 levels, leading to cell cycle arrest at the G0-G1 phase, and increased cleaved caspase-3 and cleaved caspase-9, ultimately inducing apoptosis [16].

A study revealed that CBD significantly suppressed the growth, movement, and invasion of colon cancer cells (CRC), with this effect being dose or time dependent [17]. In one study, CBD suppressed the XIAP level and induced cell death in gastric cancer cells [18].

TP53 is a crucial tumor suppressor, often referred to as the “guardian of the genome”. Transcription of pro-apoptotic genes (e.g., BAX/BIM) can be directly promoted by elevated TP53 [19]. In our study, CBD upregulated TP53, likely amplifying mitochondrial outer membrane permeabilization and caspase activation, consistent with mitochondrial apoptosis induction. In MCF-7/Adr cells, increased APAF1 and CASP9 expression suggested reactivation of the intrinsic apoptosome pathway, while FASLG induction reflected crosstalk between extrinsic and intrinsic apoptotic routes [20]. These transcriptional correlation findings collectively indicate that CBD is associated with increased expression of apoptotic mediators and counteracts survival signaling, particularly in chemoresistant cells. This is particularly relevant in the context of chemoresistance, where evasion of apoptosis is a key hallmark. Enhanced intrinsic apoptotic pathway activation in MCF-7/Adr cells suggests that CBD may overcome resistance-associated survival mechanisms.

Angiogenesis is a hallmark of tumor progression and chemoresistance, generating new vasculature from pre-existing vessels, which is essential for breast cancer growth, invasion, and metastatic dissemination, and orchestrated through a dynamic balance between pro- and anti-angiogenic mediators [21]. In this current study, CBD significantly reduced the expression of pro-angiogenic mediators, including ANGPT2, FLT1, and KDR in MCF-7 cells, as well as FLT1 and TEK (TIE2) in MCF-7/Adr cells. This dual suppression of the VEGF and ANG/TIE2 axes suggests an inhibition of both vessel formation and vascular remodeling. Such anti-angiogenic activity aligns with reports that CBD inhibits VEGF signaling and endothelial proliferation, thereby reducing tumor vascularization and metastatic potential [22,23,24].

The DNA damage response (DDR) pathways play a crucial role in maintaining genomic integrity, and their dysregulation contributes to carcinogenesis, therapy resistance, and disease progression in breast cancer [25]. Several genes are associated with DNA repair, oxidative defense, and proteostasis, including ERCC5, GADD45G, POLB, SOD1, DDIT3, LIG4, TBX2, and HSP90AB1 [26]. In our study, CBD treatment resulted in reduced levels of DDR-related genes. Additionally, unlike the MCF7/Adr cell line, a significant decrease in HSPB1 and HSP90AB4 expression was observed in MCF-7, accompanied by an increase in the apoptotic rate. The reduced expression of HSPB1 and HSP90AB4P correlated with enhanced apoptosis, supporting the notion that CBD impairs chaperone-assisted stabilization of oncogenic proteins and compromises the DNA damage response. These findings suggest that co-administration of CBD with HSP inhibitors could synergistically promote apoptosis and counteract doxorubicin resistance by turning off key cytoprotective pathways [27]. In the case of doxorubicin resistance, these defense mechanisms are frequently associated with multidrug resistance (MDR) pathways, such as increased DNA repair capacity and stress response signalling. This supports the hypothesis that CBD may interfere with resistance-associated cellular defense systems.

The primary objective of the cell cycle is to ensure precise DNA replication during the S phase and to produce two identical daughter cells during mitosis. Various checkpoints in the cell cycle ensure its proper progression. Dysregulation of these regulators is common in breast cancer, leading to uncontrolled proliferation, chromosomal instability, and resistance to therapy [28]. The results of this research suggest that CBD markedly reduced the transcription of pivotal cell-cycle regulators, including CCND2, CCND3, CDC20, E2F4, AURKA, MKI67, SKP2, and WEE1, in MCF-7 cells. This was validated by cell cycle analysis in both cell lines, which showed that CBD treatment arrested cells in the G0/G1 and G2/M phases of the cell cycle. A study of CBD treatment in three human colorectal cancer cell lines found that it arrests the cell cycle in G1, induces ER stress, and causes apoptosis [29]. CBD-induced sub-G0/G1 arrest has also been observed in several other cancer cell lines, indicating that G1 arrest is a primary mechanism for the anti-cancer activity of CBD [16,30,31]

These changes correspond to KEGG enrichment in the cell cycle and p53 signaling pathways, suggesting the enforcement of checkpoint control and the blockade of proliferation. In contrast, the upregulation of these genes in MCF-7/Adr cells may represent compensatory signaling to sustain cell division under drug stress. CBD’s modulation of checkpoint kinases and cyclins mirrors prior cannabinoid studies in gastric, glioblastoma, and breast cancer models, highlighting its context-dependent regulation of the cell cycle [16,32,33].

The process of epithelial-to-mesenchymal transition (EMT) is a significant cellular process involved in metastasis, characterized by the loss of epithelial characteristics by epithelial cells and the acquisition of a mesenchymal phenotype, resulting in increased mobility and chemoresistance [34]. In our study, CBD altered the expression of EMT genes FOXC2, SNAI2, OCLN, and GSC in MCF-7 cells, suggesting an inhibition of metastatic plasticity and partial reversion toward an epithelial phenotype. Downregulation of FOXC2 and SNAI2 may suppress migratory and invasive capabilities, while regulation of OCLN could stabilize tight junctions [35,36]. The Slug gene (SNAI2), FOXC2, and GSC are key regulators of EMT, whose expression is associated with invasion, metastasis, and chemoresistance in various cancers, including breast cancer [37,38,39]. CBD also downregulated the expression of EMT markers such as Slug and Vimentin. CBD may reduce breast cancer cell aggressiveness by inducing mesenchymal reversion to an epithelial phenotype through the relocalization of the IL-1β/IL-1R/β-catenin and E-cadherin/β-catenin complexes, as well as other protein markers of malignancy [40]. A study reveals that CBD inhibits the development of colorectal cancer (CRC) by blocking EMT and metastasis via the Wnt/β-catenin signaling pathway for the first time [17]. Our study shows that SNAI2 expression remained unchanged after CBD treatment in MCF-7/Adr cells. This suggests that SNAI2 plays a specific role in maintaining doxorubicin resistance rather than influencing the cells response to CBD [41]. EMT is associated with multidrug resistance and metastatic progression. Modulating EMT with CBD suggests a potential role in reversing aggressive and resistant phenotypes in breast cancer cells. Additionally, our results indicate that CBD inhibits the development of doxorubicin-sensitive breast cancer by blocking the EMT and metastasis processes through the TNF signaling pathway.

Additionally, CBD influenced the expression of senescence-associated genes IGFBP5, IGFBP7, MAP2K3, and TBX2, which were previously found to be downregulated in MCF-7 cells. Decreased MAP2K3 expression likely limited p38-mediated senescence and shifted the balance toward apoptosis, whereas its elevation in MCF-7/Adr might reinforce a pro-survival senescence-associated secretory phenotype (SASP). TBX2, a member of the Tbx2 subfamily of transcription factors, was overexpressed in breast cancer, which led to uncontrolled proliferation [42,43]. Downregulation of TBX2, a known anti-senescence transcription factor, further supports CBD-induced growth arrest and apoptotic priming in sensitive cells.

Telomeres are short sequences of nucleotides (TTAGG) located at the ends of chromosomes that control cellular proliferation and replication and maintain genomic integrity and stability [44]. Our study suggests that decreased expression of telomere-related genes TEP1, TERF1, and TERF2IP after CBD treatment in MCF-7 cells may limit the replication capacity of breast cancer cells by disrupting telomere activity and inhibiting telomere elongation, potentially impairing telomerase function and triggering telomere shortening. This mechanism can activate p53-dependent senescence or apoptosis [45,46,47,48]. In MCF-7/Adr cells, upregulation of TERF2IP may be an adaptive response to maintain genomic stability under stress. This indicates different telomeric responses to CBD between sensitive and resistant phenotypes [49].

Glycolysis, the primary energy-generating pathway that converts glucose to pyruvate, is increased in cancer cells due to the Warburg effect, thereby supporting their survival. In our study, CBD treatment altered the energy metabolism of both MCF-7 and MCF-7/Adr cells. The suppression of PFKL, LDHA, and SLC2A1 gene expression in MCF-7 cells inhibited glycolysis and glucose uptake, thereby reversing the Warburg effect and reducing bioenergetic capacity. Conversely, their upregulation in MCF-7/Adr cells indicated a response involving increased metabolic activity to support survival, which is consistent with the enhanced glucose flux and mitochondrial respiration that resistant cells rely on. Tumor cells can use fatty acids as an alternative energy source. They play a crucial role in supporting the metabolic needs of cancer cells [50]. The upregulation of fatty acid uptake and synthesis facilitates tumor cell proliferation and migration. Currently, limited information is available regarding the relationship between CBD-regulated lipid metabolism and apoptosis; however, further research is necessary to explore this area [51].

In our study, CBD treatment resulted in a significant reduction in the expression of ACLY, ACSL4, and CPT2 in MCF-7 cells, suggesting an inhibition of fatty acid synthesis, lipid activation, and fatty acid oxidation. Previous studies have demonstrated that CBD treatment reduces intracellular lipid accumulation in various cell types [52,53]. Furthermore, gene–metabolite analysis revealed the presence of glycolysis-related metabolites, including fructose-6-phosphate, glucose-6-phosphate, dihydroxyacetone phosphate, β-D-glucose-6-phosphate, ATP, and ADP. The detection of these metabolites indicates that multiple steps of glycolysis were affected, reflecting a reprogramming of energy production pathways. Previous studies have shown that CBD enhances glucose uptake in adipocytes while also suppressing the activities of fructose-1,6-bisphosphatase, glucose-6-phosphatase, glycogen phosphorylase, and lipase [54]. The results of these transcriptional analyses indicate that CBD induces metabolic stress in sensitive cells, highlighting weaknesses in resistant phenotypes and strongly suggesting that CBD may initiate apoptosis by disrupting glucose and lipid metabolism.

KEGG pathway enrichment analysis provided valuable insights at a systems level, demonstrating that both MCF-7 and MCF-7/Adr cells exhibited significant enrichment in the p53, HIF-1, PI3K-Akt, MAPK, RAP1, and RAS signaling pathways. Additionally, pathways associated with apoptosis, cellular senescence, and transcriptional dysregulation in cancer were identified. In MCF-7, the activation of stress and checkpoint networks was highlighted by additional enrichment in TNF signaling and cell cycle regulation. In contrast, in MCF-7/Adr, metabolic reprogramming was emphasized as a central feature of resistance, as indicated by enrichment in central carbon metabolism in cancer. These integrated signatures confirm that CBD exerts multiple inhibitory effects on oncogenic signaling by simultaneously targeting growth, survival, angiogenesis, DNA repair, and metabolism. Many pathways are implicated in multidrug resistance. These pathways also regulate the expression and activity of ATP-binding cassette (ABC) transporters. ABCB1 (P-glycoprotein), ABCC1, and ABCG2 contribute to doxorubicin resistance by exporting the drug out of cancer cells. Although ABC transporter activity was not directly assessed in our study, the observed transcriptional modulation of survival and stress-response pathways suggests that CBD may indirectly influence these resistance mechanisms. Simultaneously suppressing the PI3K-Akt, MAPK, and RAS cascades reduces the activity of transcription factors that promote survival, while activating p53-dependent apoptosis and restricting metabolism weakens tumor maintenance. This multi-pathway disruption is consistent with the concept of polypharmacology, where CBD disrupts multiple cancer hallmarks at the same time rather than through a single target [55,56].

Overall, the data depict CBD as a multitarget antineoplastic and chemosensitizing agent that can reverse resistance-associated phenotypes. These findings support the emerging evidence that cannabinoids can increase the sensitivity of cancer cells to chemotherapy and modulate resistance-related pathways. By concurrently inhibiting angiogenesis, suppressing DNA repair and chaperone networks, activating apoptotic cascades, impairing metabolic flexibility, and altering telomere and EMT programs, CBD orchestrates a coordinated transcriptional response that dismantles tumor robustness. The downregulation of PI3K-Akt, MAPK, and HIF-1 signaling pathways, which are often implicated in therapy resistance, suggests that CBD could enhance the efficacy of doxorubicin or other standard treatments. Future studies should explore the translational potential of CBD in combination treatments, verifying these transcriptomic effects at proteomic and functional levels in vivo.

This study has limitations that should be considered when interpreting the findings. First, all experiments were conducted exclusively in vitro using MCF-7 and MCF-7/Adr cell lines. While these models provide valuable insight into doxorubicin sensitivity and resistance, they do not fully replicate the complexity of the tumor microenvironment, including immune interactions, stromal components, vascular architecture, hypoxia, and drug metabolism present in vivo. Therefore, the antitumor and chemosensitizing effects of CBD observed here may not directly translate to animal models or clinical settings. Second, molecular analyses were mainly at the transcriptional level, with no protein-level validation. Future studies should confirm these findings at the protein level.

Also, functional studies were limited to viability, clonogenicity, invasion, apoptosis, and cell-cycle assays. Third, although CBD is discussed as a possible chemosensitizing agent, experiments with DXR were not performed. Future studies should include CBD-DXR co-treatment models, particularly in resistant cell lines. The study did not investigate mechanistic causality, such as whether CBD directly modulates specific signaling nodes (e.g., PI3K/AKT, MAPK, p53) or whether the observed gene expression changes are secondary effects. Knockdown or inhibition studies would be required to confirm mechanistic pathways. Finally, the study did not examine potential long-term effects, off-target toxicity, or interactions between CBD and doxorubicin in combination treatments. These gaps highlight the need for further studies.

## 4. Materials and Methods

### 4.1. Cell Culture and Treatment

In this study, human doxorubicin-sensitive and doxorubicin-resistant breast cancer cell lines, MCF-7 (ATCC, HTB-22) and MCF-7/Adr (iCell, HTB-22), along with the normal human fibroblast cell line CCD-1072Sk(ATCC), were used. These cell lines are widely used in cancer research due to their relevance in studying drug resistance and sensitivity in breast cancer. The MCF7, MCF-7/Adr and CCD-1072sk cell lines were cultured in DMEM medium (Gibco, Thermo Fisher, Waltham, MA, USA) supplemented with 10% FBS, 1% pen/strep, and 1% L-glutamine in a humidified incubator at 37 °C with 5% CO_2_ and 95% humidity. The MCF7/Adr cell line was exposed to 5 µM doxorubicin 2–3 times. Cannabidiol (CBD), purchased from CBD Depot, was used in the experiments after being formulated from a 20 mM stock in 0.1% dimethyl sulfoxide (DMSO) and stored at −20 °C until used.

### 4.2. Cell Viability

The effects of CBD on the viability of MCF-7, MCF-7/Adr and CCD-1072sk cells were assessed using the MTT assay. This assay measures the number of live cells in a sample by determining their metabolic activity. The cells were cultured under optimal growth conditions, seeded into 96-well plates at a density of 5 × 10^3^ cells per well, and incubated until sufficient confluency was achieved. CBD was administered at concentrations ranging from 0 to 200 μM (200, 100, 50, 25, 12.5, 6.25, 3.125, 1.56 μM) at 24, 48, and 72 h. At the end of the experiment, the IC50 value of CBD (the concentration that reduces cell viability by 50%) was calculated using the absorbance readings obtained with an ELISA plate reader. The percentages for each dose and the viable control group were determined using the appropriate formula.

### 4.3. Colony Assay

The colony formation assay was employed to investigate the inhibitory effects of CBD on the proliferative capacity of MCF-7 and MCF-7/Adr breast cancer cells. This test is instrumental in assessing the long-term clonogenic survival of cells post-treatment. In this assay, MCF-7 and MCF-7/Adr cells were plated at a density of 2 × 10^3^ cells per well in six-well plates. Following a 24 h period to allow for cell attachment at 37 °C, the cells were treated with CBD. Subsequently, the culture medium was refreshed bi-daily. After 12 days, cells were gently rinsed with PBS, fixed with 100% cold methanol at −20 °C for 10 min, and stained with a 0.5% crystal violet solution for 15 min to visualize colonies. CBD doses and control group colonies were photographed under a microscope. Colony numbers in the control and dose groups were manually counted in three different wells, and their averages were calculated. The number of colonies in the control and dose groups was manually counted in three different wells, and the average was calculated.

### 4.4. Invasion Assay

To determine the effect of CBD on the invasive behavior of MCF-7 and MCF-7/Adr breast cancer cells, an invasion assay was carried out using the Corning^®^ BioCoat™ Matrigel^®^ Invasion Chamber (Corning (Corning, NY, USA), #354480). This test was crucial in determining whether CBD could prevent these cells from proliferating. Cells from control and CBD-treated groups were resuspended in serum-free medium at a density of 2.5 × 10^5^ cells/mL to eliminate serum-derived migratory stimuli. The upper chamber was filled with 500 μL of this mixture. The lower chamber was filled with complete culture medium supplemented with 10% fetal bovine serum (FBS), which served as a chemoattractant. Conversely, cells that migrated to the lower side were fixed with 100% methanol and subsequently stained with 0.1% crystal violet to ensure optimal visualization. The effects of CBD on cell invasion were assessed by counting the invasive cells in three randomly selected areas under a light microscope.

### 4.5. PCR Array Studies

Real-time quantitative PCR (RT-qPCR) was used to investigate the effects of CBD on cancer-related processes. For RNA isolation, MCF-7 and MCF-7/Adr cells were seeded at a density of 3 × 10^5^ cells per well in 6-well plates. After 24 h of incubation, the CBD was applied at its predetermined IC50 concentrations. Following 48 h of compound treatment, total RNA was isolated using the innuPREP RNA Mini Kit 2.0 (Innuscreen GmbH, Berlin, Germany), and cDNA synthesis was performed with the Wonder RT-cDNA Kit (Euroclone, Pero, Italy), both according to the manufacturers’ instructions. Cancer array gene set primers (Supplementary Table S1) (Merck, Darmstadt, Germany) were used, with ACTB and GAPDH as reference genes. RT-qPCR was performed with the FluoCycle II SYBR Master Mix (Euroclone, Pero, Italy) according to the manufacturer’s instructions. The PCR reaction conditions were as follows: an initial denaturation at 95 °C for 5 min, followed by 45 cycles of 15 s at 95 °C and 30 s at 60 °C. The results were analyzed using the method described previously [57]. RT-qPCR analysis was performed based on three replicates. Therefore, fold-change values are presented descriptively. Genes with significant expression alterations (≥2-fold increase or ≤0.5-fold decrease) were selected, and KEGG pathway analysis was performed using the ShinyGO 0.82 software [58,59,60].

### 4.6. Detection of Apoptosis

The use of the FITC Annexin V apoptosis detection assay was crucial for elucidating the pro-apoptotic potential of CBD in MCF-7 and MCF-7/Adr breast cancer cells, providing insights into its therapeutic efficacy against cellular proliferation by inducing programmed cell death. The apoptotic effects of CBD on MCF-7 and MCF-7/Adr breast cancer cells were quantified using flow cytometry with the Annexin V-FITC Apoptosis Detection Kit (Sigma-Aldrich, St. Louis, MO, USA), and the PI staining technique was used to show the apoptotic effect of the agents applied to the cell lines. MCF-7 and MCF-7/Adr cells were plated at a density of 3 × 10^5^ cells/per well in 6-well plates. After 24 h of incubation, CBD was administered at the predetermined IC_50_ concentrations. After 48 h of compound treatment, the cells were centrifuged, the binding buffer was added, and the homogenate was prepared for the assay. An aliquot of 100 μL homogenized cells and 5 μL of Annexin V were incubated for 15 min at 25 °C in the dark, then analyzed by flow cytometry. As Annexin V binding can also be observed on the surface of necrotic cells, 5 μL of propidium iodide was added as a second dye to the flow cytometry [61].

### 4.7. Detection of Cell Cycle

Breast cancer cells were seeded at 3 × 10^5^ cells per well into 6-well plates and incubated at 37 °C, 5% CO_2_, and 95% humidity for 24 h. After 24 h, the specified CBD IC_50_ concentration was added to one well per cell line, and fresh medium was added to the untreated control group. The plates were incubated for 48 h, and analysis was performed according to the instructions for the MAK344 Cell Cycle Analysis kit (Sigma-Aldrich, Burlington, MA, USA).Then, the cells were analyzed using flow cytometry (Beckman Coulter, Cytoflex, Indianapolis, IN, USA) [61].

### 4.8. Gene-Metabolite Interaction

Gene expressions show significant differences, and their related metabolites were analyzed using the Enrichr tool with the Metabolomics Workbench Metabolites 2022 database [62,63]. The interaction network between the identified metabolites and genes was constructed using MetaboAnalyst 6.0 [64].

### 4.9. Statistical Analysis

All experiments were performed at least in triplicate, and data are presented as mean ± standard deviation (SD). Statistical analyses were conducted using GraphPad Prism version 8.02 (GraphPad Software, San Diego, CA, USA). Comparisons between two groups were performed using an unpaired two-tailed Student’s *t*-test. In contrast, multiple group comparisons were analyzed using one-way analysis of variance (ANOVA) followed by Tukey’s post hoc test. A *p*-value < 0.05 was considered statistically significant.

## 5. Conclusions

This study shows that cannabidiol (CBD) exerts multitarget antitumor and chemosensitizing effects in doxorubicin-sensitive (MCF-7) and doxorubicin-resistant (MCF-7/Adr) breast cancer cells. CBD reduced viability, proliferation, clonogenicity, and invasion while inducing apoptosis, and modulated the transcription of key genes involved in apoptosis, angiogenesis, DNA-damage response, cell cycle regulation, EMT, telomere maintenance, and energy metabolism. These changes indicate that CBD disrupts multiple cancer hallmarks and counteracts resistance-associated adaptations, particularly in the MCF-7/Adr phenotype. Overall, our findings support CBD as a promising multitarget candidate for breast cancer therapy, especially for chemoresistance.

## Data Availability

The original contributions presented in this study are included in the article/Supplementary Material. Further inquiries can be directed to the corresponding author.

## Supplementary Materials

The following supporting information can be downloaded at https://www.mdpi.com/article/10.3390/ph19040615/s1, Table S1: List of primers used in this study; Table S2: RT-qPCR results obtained following cannabidiol treatment in MCF-7 cells; Table S3: RT-qPCR results obtained following cannabidiol treatment in MCF-7/Adr cells; Table S4: Genes exhibiting significant expression changes in MCF-7 cells following RT-qPCR analysis (≥2-fold upregulation or ≤0.5-fold downregulation) were identified and gene enrichment analysis was performed using the ShinyGO program; Table S5: Genes exhibiting significant expression changes in MCF-7/Adr cells following RT-qPCR analysis (≥2-fold upregulation or ≤0.5-fold downregulation) were identified and gene enrichment analysis was performed using the ShinyGO program; Table S6: Genes exhibiting significant expression changes in MCF-7 cells following RT-qPCR analysis (≥2-fold upregulation or ≤0.5-fold downregulation) were identified and their associated metabolites were determined using the EnrichR platform with the Metabolomics Workbench Metabolites 2022 database (accessed 30 June 2025); Table S7: Genes exhibiting significant expression changes in MCF-7/Adr cells following RT-qPCR analysis (≥2-fold upregulation or ≤0.5-fold downregulation) were identified and their associated metabolites were determined using the EnrichR platform with the Metabolomics Workbench Metabolites 2022 database (accessed 30 June 2025).

## Abbreviations

The following abbreviations are used in this manuscript:

CBD	Cannabidiol
ADR	Adrymicin
DXR	Doxorubicin
