# Supplementary material for: The Effect of Cannabidiol on Cancer-Pathway Genes in Doxorubicin-Sensitive and Resistant Breast Cancer Cells
Source: Pharmaceuticals (Basel). 2026 Apr 14;19(4):615. doi: 10.3390/ph19040615 (PMC13119141; doi:10.3390/ph19040615)
Supplement: Supplementary file 1 [file pharmaceuticals-19-00615-s001.zip › pharmaceuticals-4209654-supplementary.pdf]

Supplementary File

The Effect of Cannabidiol on Cancer-Pathway Genes in Doxorubicin-Sensitive and Resistant Breast Cancer Cells

Supplementary Table S1: List of primers used in this study.

ACLY	*BIRC3*	*CDC37*	*E2F4*	*GADD45G*	*IGFBP7*	*MKI67*	*SKP2*	*TEP1*	*WEE1*
ACSL4	*BMI1*	*CDH2*	*EPO*	*GPD2*	*KDR*	*NOL3*	*SLC2A1*	*TERF1*	*XIAP*
ADM	*CA9*	*CDK4*	*ERCC3*	*GSC*	*KRT14*	*OCLN*	*SNAI1*	*TERF2IP*	*GAPDH*
ANGPT1	*CASP2*	*CFLAR*	*ERCC5*	*GUSB*	*LDHA*	*PFKL*	*SNAI2*	*TINF2*	*ACTB*
ANGPT2	*CASP7*	*COX5A*	*ETS2*	*HMOX1*	*LIG4*	*PGF*	*SNAI3*	*TNKS*	
APAF1	*CASP9*	*CPT2*	*FASLG*	*HSP90AB1*	*LPL*	*PINX1*	*SOD1*	*TNKS2*	
ARNT	*CCL2*	*DDB2*	*FGF2*	*HSP90AB4P*	*MAP2K1*	*POLB*	*SOX10*	*TP53*	
ATP5A1	*CCND2*	*DDIT3*	*FLT1*	*HSPB1*	*MAP2K3*	*PPP1R15A*	*STMN1*	*TRAP1*	
AURKA	*CCND3*	*DKC1*	*FOXC2*	*IGFBP3*	*MAPK14*	*SERPINB2*	*TBX2*	*UQCRFS1*	
BCL2L11	*CDC20*	*DSP*	*G6PD*	*IGFBP5*	*MCM2*	*SERPINF1*	*TEK*	*VEGFC*	

Supplementary Table S2: RT-qPCR results obtained following cannabidiol treatment in MCF-7 cells. All experiments were conducted using three independent biological replicates (n = 3). Data are expressed as mean ± standard deviation (SD). Fold changes were calculated using the 2^−ΔΔCt method, with the control group normalized to 1. Statistical significance was defined as *p < 0.05, **p < 0.01, and ***p < 0.001.

Gene	Fold Change	Gene	Fold Change	Gene	Fold Change
ACLY	0.60 ± 0.7	*EPO*	0.10 ± 0.18*	*OCLN*	0.05 ±0.07*
ACSL4	0.41 ± 0.52	*ERCC3*	0.57 ± 0.75	*PFKL*	0.32 ± 0.44
ADM	0.56 ± 0.82	*ERCC5*	0.33 ± 0.45	*PGF*	1.15 ± 2.01
ANGPT1	0.03 ± 0.1*	*ETS2*	0.82 ± 1.19	*PINX1*	0.64 ± 0.85
ANGPT2	0.09 ± 0.16	*FASLG*	0.02 ± 0.05**	*POLB*	0.35 ± 0.42
APAF1	1.04 ± 1.56	*FGF2*	0.24 ± 0.3	*PPP1R15A*	0.72 ± 1.12
ARNT	0.46 ± 0.65	*FLT1*	0.04 ± 0.1*	*SERPINB2*	0.39 ± 0.61
ATP5A1	1.11 ± 1.13	*FOXC2*	0.22 ± 0.36	*SERPINF1*	0.48 ± 0.91
AURKA	0.46 ± 0.53	*G6PD*	1.54 ± 2.16	*SKP2*	0.37 ± 0.49
BCL2L11	0.31 ± 0.46	*GADD45G*	0.07 ± 0.14**	*SLC2A1*	0.95 ± 1.21

BIRC3	0.35 ± 0.37	*GPD2*	0.37 ± 0.51	*SNAI1*	0.56 ± 1.02
BMI1	0.54 ± 0.7	*GSC*	0.38 ± 0.59	*SNAI2*	0.34 ± 0.62
CA9	0.25 ± 0.25	*GUSB*	0.22 ± 0.24	*SNAI3*	1.05 ±2.12
CASP2	0.79 ± 1.32	*HMOX1*	0.84 ± 1.09	*SOD1*	0.26 ±0.25
CASP7	0.78 ± 1.05	*HSP90AB1*	0.52 ± 0.48	*SOX10*	0.54 ±1
CASP9	0.65 ± 0.93	*HSP90AB4P*	0.30 ± 0.39	*STMN1*	1.56 ± 2.68
CCL2	0.03 ± 0.05**	*HSPB1*	1.09 ± 2.58	*TBX2*	0.03 ± 0.05**
CCND2	0.08 ± 0.13*	*IGFBP3*	0.52 ± 0.73	*TEK*	1.14 ±2.63
CCND3	0.43 ± 0.64	*IGFBP5*	0.02 ± 0.04**	*TEP1*	0.40 ± 0.56
CDC20	0.91 ± 1.01	*IGFBP7*	0.23 ± 0.36	*TERF1*	0.33 ± 0.38
CDC37	1.24 ± 2.18	*KDR*	0.39 ± 0.7	*TERF2IP*	0.28 ± 0.34
CDH2	0.53 ± 0.63	*KRT14*	1.85 ± 3.12	*TINF2*	0.42 ± 0.51
CDK4	0.70 ± 0.74	*LDHA*	0.43 ± 0.37	*TNKS*	0.50 ± 0.67
CFLAR	0.41 ± 0.57	*LIG4*	0.03 ± 0.05**	*TNKS2*	0.56 ± 0.72
COX5A	2.40 ± 2.99	*LPL*	0.38 ± 0.47	*TP53*	1.06 ± 1.4
CPT2	0.25 ± 0.36	*MAP2K1*	0.53 ± 0.64	*TRAP1*	1.31 ± 1.6
DDB2	0.79 ± 1.07	*MAP2K3*	0.12 ± 0.15*	*UQCRFS1*	0.41 ± 0.44
DDIT3	0.35 ± 0.41	*MAPK14*	0.61 ± 0.77	*VEGFC*	0.62 ± 0.78
DKC1	1.48 ± 1.99	*MCM2*	1.22 ± 1.42	*WEE1*	0.40 ± 0.52
DSP	1.15 ± 1.17	*MKI67*	0.36 ± 0.45	*XIAP*	0.48 ± 0.62
E2F4	0.24 ± 0.33	*NOL3*	1.24 ± 1.91		

Supplementary Table S3: RT-qPCR results obtained following cannabidiol treatment in MCF-7/Adr cells. All experiments were conducted using three independent biological replicates (n = 3). Data are expressed as mean ± standard deviation (SD). Fold changes were calculated using the 2^−ΔΔCt method, with the control group normalized to 1. Statistical significance was defined as *p < 0.05, **p < 0.01, and ***p < 0.001.

**Gene**	**Fold Change**	**Gene**	**Fold Change**	**Gene**	**Fold Change**
ACLY	1.45 ± 0.04*	*EPO*	2.44 ± 0.26	*OCLN*	1.76 ± 0.08**
ACSL4	1.87 ± 0.08**	*ERCC3*	1.45 ± 0.68	*PFKL*	2.72 ± 0.4**
ADM	1.11 ± 0.13	*ERCC5*	2.65 ± 0.24	*PGF*	3.69 ± 0.37
ANGPT1	1.37 ± 0.15*	*ETS2*	3.15 ± 0.97	*PINX1*	1.98 ± 0.96*
ANGPT2	1.55 ± 0.21*	*FASLG*	2.14 ± 0.39	*POLB*	1.62 ± 0.06**
APAF1	2.27 ± 0.23**	*FGF2*	0.89 ± 1.08	*PPP1R15A*	48.00 ± 2.27***

ARNT	1.03 ± 0.13	*FLT1*	0.92 ± 0.13	*SERPINB2*	1.80 ± 1.96
ATP5A1	1.21 ± 0.01	*FOXC2*	1.62 ± 0.18**	*SERPINF1*	5.92 ± 1.28**
AURKA	1.43 ± 0.01*	*G6PD*	3.11 ± 0.38**	*SKP2*	1.61 ± 1.63*
BCL2L11	1.55 ± 0.15*	*GADD45G*	1.24 ± 0.17*	*SLC2A1*	3.35 ± 1.28*
BIRC3	2.03 ± 0.09**	*GPD2*	2.15 ± 1.08	*SNAI1*	1.69 ± 0.47
BMI1	1.41 ± 0.18*	*GSC*	1.30 ± 0.14*	*SNAI2*	3.82 ± 0.1**
CA9	1.29 ± 0.25	*GUSB*	1.37 ± 1.84	*SNAI3*	1.63 ± 0.07
CASP2	1.81 ± 0.1**	*HMOX1*	2.63 ± 0.97	*SOD1*	1.15 ± 0.38
CASP7	1.33 ± 0.98*	*HSP90AB1*	1.72 ± 0.57*	*SOX10*	1.02 ± 0.45
CASP9	2.67 ± 0.28**	*HSP90AB4P*	1.49 ± 0.84*	*STMN1*	1.31 ± 0.66
CCL2	0.94 ± 0.1	*HSPB1*	36.13 ± 5.32**	*TBX2*	2.15 ± 1.08**
CCND2	6.08 ± 0.68***	*IGFBP3*	2.39 ± 0.97	*TEK*	0.16 ± 0.6***
CCND3	1.28 ± 0.45*	*IGFBP5*	2.32 ± 1.08	*TEP1*	1.66 ± 0.37
CDC20	2.20 ± 0.1**	*IGFBP7*	0.11 ± 0.33	*TERF1*	1.24 ± 0.51
CDC37	20.32 ± 3.83***	*KDR*	2.54 ± 0.87**	*TERF2IP*	2.67 ± 1.08*
CDH2	3.47 ± 0.11**	*KRT14*	7.09 ± 1.48	*TINF2*	1.50 ± 0.63
CDK4	2.09 ± 0.28**	*LDHA*	1.62 ± 0.97*	*TNKS*	1.83 ± 0.84
CFLAR	1.40 ± 0.97*	*LIG4*	1.44 ± 0.45	*TNKS2*	1.54 ± 0.63
COX5A	40.64 ± 3.01***	*LPL*	1.81 ± 0.67	*TP53*	0.55 ± 0.44**
CPT2	5.01 ± 0.4	*MAP2K1*	1.44 ± 1.27	*TRAP1*	2.39 ± 1.14*
DDB2	1.77 ± 1.14**	*MAP2K3*	2.37 ± 1.67	*UQCRFS1*	1.24 ± 0.67
DDIT3	0.03 ± 0.8**	*MAPK14*	1.15 ± 0.63	*VEGFC*	1.49 ± 0.96
DKC1	7.49 ± 1.45	*MCM2*	1.50 ± 0.3*	*WEE1*	1.78 ± 0.48
DSP	1.30 ± 0.07	*MKI67*	1.54 ± 0.84*	*XIAP*	2.09 ± 1.07
E2F4	1.76 ± 1.2**	*NOL3*	3.69 ± 1.63		

Supplementary Table S4: Genes exhibiting significant expression changes in MCF-7 cells following RT-qPCR analysis (≥2-fold upregulation or ≤0.5-fold downregulation) were identified and gene enrichment analysis was performed using the ShinyGO program. Related pathways/events were selected based on FDR values and ranked according to fold enrichment scores (accessed June 30, 2025). Enrichment FDR represents the false discovery rate–adjusted p-value (Benjamini–Hochberg correction) indicating the statistical significance of pathway enrichment. nGenes refers to the number of input genes associated with each pathway, while Pathway Genes indicates the total number of genes annotated for the corresponding pathway in the reference database. Fold enrichment

represents the ratio of observed to expected gene counts, reflecting the degree of pathway overrepresentation.

Pathways	Enrichment FDR	nGenes	Pathway Genes	Fold Enrichment
Apoptosis multiple species	3.4E-05	4	32	38.1
p53 signalling pathway	9.3E-08	7	73	29.3
HIF-1 signalling pathway	4.3E-09	9	109	25.2
Apoptosis	5.1E-11	11	136	24.7
Small cell lung cancer	3.9E-07	7	92	23.2
TNF signalling pathway	8.2E-08	8	112	21.8
Cell cycle	1.7E-07	8	126	19.4
Cellular senescence	6.1E-08	9	156	17.6
FoxO signalling pathway	3.1E-06	7	131	16.3
EPV infection	3.8E-08	10	202	15.1
Non-alcoholic fatty liver disease	8.1E-06	7	155	13.8
Rap1 signalling pathway	4.8E-07	9	210	13.1
PI3K/AKT signalling pathway	5.1E-11	15	354	12.9
Transcriptional misregulation in cancer	3.1E-06	8	193	12.6
MAPK signalling pathway	8.2E-09	12	294	12.5
Human T-cell leukemia virus 1 infection	7.1E-07	9	222	12.4
Focal adhesion	3.5E-06	8	200	12.2
Ras signalling pathway	1.1E-06	9	235	11.7
Pathways in cancer	6.8E-14	20	530	11.5
Lipid and atherosclerosis	5.6E-06	8	214	11.4

Supplementary Table S5: Genes exhibiting significant expression changes in MCF-7/Adr cells following RT-qPCR analysis (≥2-fold upregulation or ≤0.5-fold downregulation) were identified and gene enrichment analysis was performed using the ShinyGO program. Related pathways/events were selected based on FDR values and ranked according to fold enrichment scores (accessed June 30,

2025). Enrichment FDR represents the false discovery rate-adjusted p-value (Benjamini–Hochberg correction) indicating the statistical significance of pathway enrichment. nGenes refers to the number of input genes associated with each pathway, while Pathway Genes indicates the total number of genes annotated for the corresponding pathway in the reference database. Fold enrichment represents the ratio of observed to expected gene counts, reflecting the degree of pathway overrepresentation.

Pathways	Enrichment FDR	nGenes	Pathway Genes	Fold Enrichment
Apoptosis multiple species	4.2E-06	4	32	66.5
Platinum drug resistance	1.4E-07	6	73	43.7
p53 signalling pathway	1.4E-07	6	73	43.7
Small cell lung cancer	4.0E-07	6	92	34.7
Central carbon metabolism in cancer	7.3E-05	4	70	30.4
HIF-1 signalling pathway	9.7E-07	6	109	29.3
Apoptosis	1.5E-07	7	136	27.4
Measles	3.0E-06	6	139	23
MAPK signalling pathway	6.7E-09	10	294	18.1
PI3K/AKT signalling pathway	2.3E-10	12	354	18
Lipid and atherosclerosis	2.2E-06	7	214	17.4
Cellular senescence	7.7E-05	5	156	17.1
Human T-cell leukemia virus 1 infection	2.6E-06	7	222	16.8
Transcriptional misregulation in cancer	1.6E-05	6	193	16.5
Focal adhesion	1.8E-05	6	200	16
Ras signalling pathway	3.1E-06	7	235	15.9
EBV infection	1.8E-05	6	202	15.8
Rap1 signalling pathway	2.2E-05	6	210	15.2
Pathways in cancer	6.7E-10	13	530	13.1
Parkinson disease	7.6E-05	6	266	12

Supplementary Table S6: Genes exhibiting significant expression changes in MCF-7 cells following RT-qPCR analysis (≥2-fold upregulation or ≤0.5-fold downregulation) were identified and their associated metabolites were determined using the EnrichR platform with the Metabolomics Workbench Metabolites 2022 database (accessed 30 June 2025).

Index	Name	P-value	Adjusted p-value	Odds Ratio	Combined score
1	Palmitoyl-CoA	0.002337	0.05821	31.65	191.76
2	Coenzyme A	0.006369	0.05821	8.49	42.92
3	ATP	0.006985	0.05821	5.62	27.90
4	3-Mercaptopyruvic Acid	0.02259	0.06576	53.12	201.33
5	AMP	0.02468	0.06576	8.66	32.05
6	Triacylglycerol	0.02630	0.06576	44.26	161.03
7	Dihydroxyacetone Phosphate	0.02630	0.06576	44.26	161.03
8	Palmitic Acid	0.02630	0.06576	44.26	161.03
9	Fructose 1.6-Bisphosphate	0.02630	0.06576	44.26	161.03
10	Glycerol	0.02630	0.06576	44.26	161.03
11	sn-Glycero-3-phosphate	0.03000	0.06819	37.94	133.02
12	3-Methyl Pyruvic Acid	0.03369	0.07019	33.19	112.54
13	FAD	0.04830	0.08113	22.12	67.04
14	CDP	0.05192	0.08113	20.42	60.41
15	UTP	0.05192	0.08113	20.42	60.41
16	ITP	0.05192	0.08113	20.42	60.41
17	Fructose 6-Phosphate	0.05553	0.08166	18.96	54.82
18	IDP	0.05912	0.08211	17.70	50.05
19	CTP	0.06626	0.08719	15.61	42.38
20	Glucuronic Acid	0.07335	0.09152	13.97	36.49
21	Pyruvic Acid	0.07688	0.09152	13.27	34.04
22	ADP	0.09221	0.1048	4.05	9.66
23	Acetyl-CoA	0.1249	0.1357	7.80	16.23
24	UDP	0.2014	0.2098	4.57	7.32
25	NAD+	0.3353	0.3353	2.49	2.72

Supplementary Table S7: Genes exhibiting significant expression changes in MCF-7/Adr cells following RT-qPCR analysis (≥2-fold upregulation or ≤0.5-fold downregulation) were identified and their associated metabolites were determined using the EnrichR platform with the Metabolomics Workbench Metabolites 2022 database (accessed 30 June 2025).

**Index**	**Name**	**P-value**	**Adjusted p-value**	**Odds Ratio**	**Combined score**
1	Fructose 1.6-Bisphosphate	0.01461	0.05515	81.11	342.76
2	Dihydroxyacetone Phosphate	0.01461	0.05515	81.11	342.76
3	sn-Glycero-3-phosphate	0.01668	0.05515	69.52	284.57
4	Glucose 6-Phosphate	0.02081	0.05515	54.06	209.35
5	alpha-D-Glucose 6-Phosphate	0.02081	0.05515	54.06	209.35
6	beta-D-Glucose 6-Phosphate	0.02081	0.05515	54.06	209.35
7	FAD	0.02697	0.05515	40.54	146.48
8	CDP	0.02901	0.05515	37.42	132.47
9	UTP	0.02901	0.05515	37.42	132.47
10	ITP	0.02901	0.05515	37.42	132.47
11	Fructose 6-Phosphate	0.03105	0.05515	34.75	120.64
12	IDP	0.03309	0.05515	32.43	110.53
13	CTP	0.03715	0.05596	28.61	94.21
14	Palmitoyl-CoA	0.03917	0.05596	27.02	87.54
15	UDP	0.1168	0.1557	8.37	17.97
16	Coenzyme A	0.1883	0.2215	4.94	8.25
17	NADP+	0.1883	0.2215	4.94	8.25
18	NAD+	0.2019	0.2243	4.57	7.31
19	ADP	0.2462	0.2592	3.64	5.10
20	ATP	0.3432	0.3432	2.43	2.60
